# In Vivo PET Imaging of [^18^F]CHDI-385, a Radioligand for Mutant Huntingtin Aggregates in a Mouse Model of Huntington Disease

**DOI:** 10.2967/jnumed.125.270660

**Published:** 2026-02

**Authors:** Franziska Zajicek, Filipe Elvas, Alan Miranda, Jordy Akkermans, Jeroen Verhaeghe, Celia Dominguez, Robert Doot, Vinod Khetarpal, Jonathan Bard, Longbin Liu, Steven Staelens, Daniele Bertoglio

**Affiliations:** 1 Molecular Imaging Center Antwerp, University of Antwerp, Antwerp, Belgium;; 2 *µ*Neuro Research Center of Excellence, University of Antwerp, Antwerp, Belgium;; 3 Department of Nuclear Medicine, Antwerp University Hospital, Antwerp, Belgium;; 4 CHDI Management, Los Angeles, California; and; 5 Bio-Imaging Laboratory, University of Antwerp, Antwerp, Belgium

**Keywords:** Huntington disease, zQ175DN, mHTT, [^18^F]CHDI-385, huntingtin

## Abstract

Aggregation of mutant huntingtin (mHTT) is a neurologic hallmark of Huntington disease (HD), a neurodegenerative disorder caused by the expansion of a cytosine–adenine–guanine repeat tract in the huntingtin gene (*HTT*). With a considerable number of candidate therapeutic interventions aimed at lowering mHTT expression under investigation, noninvasive monitoring of changes in mHTT aggregate levels in the brain could hasten the development and identification of disease-modifying therapies. Here we evaluate a new radioligand, [^18^F]CHDI-385, to quantify mHTT aggregates using microPET imaging in the zQ175DN mouse model of HD. **Methods:** In 3- and 9-mo old wild-type (*n* = 24 for each age) and heterozygous zQ175DN (*n* = 24 for each age) mice, we assessed the plasma and brain radiometabolite profile, explored in vivo tracer kinetics (including test–retest variability), and performed quantitative (using total volume of distribution based on a noninvasive image-derived input function, 0–120 min) and semiquantitative (using SUV; time interval, 100–120 min after injection) analyses to determine the performance of this radioligand in detecting mHTT aggregates in vivo. **Results:** [^18^F]CHDI-385 showed metabolic stability in both wild-type and heterozygous mice as well as sufficient cerebral retention time in both genotypes. Quantitative (2-tissue compartmental model and Logan graphical analysis) and semiquantitative (SUV) analyses were in strong agreement with one another (striatum, *r^2^* = 0.986; *P* < 0.0001). Differences in measures of [^18^F]CHDI-385 uptake were significant between heterozygous mice and wild-type mice at both 3 mo (*P* < 0.001) and 9 mo (*P* < 0.0001). In addition, [^18^F]CHDI-385 displayed a good to excellent test–retest variability as indicated by the intraclass correlation coefficient (ICC) with both quantitative (ICC, 0.62–0.78) and semiquantitative (ICC, 0.65–0.80) analyses. **Conclusion:** [^18^F]CHDI-385 demonstrated excellent kinetics and reliable semiquantitative and quantitative performance. Importantly, the validation of semiquantitative analysis supports the use of the more clinically friendly SUV metric, which does not require the use of an input function and metabolite correction. These results indicate that [^18^F]CHDI-385 is a radioligand with optimal properties for detecting and quantifying cerebral mHTT aggregates and support its clinical evaluation.

## Visual Abstract

**Figure:**
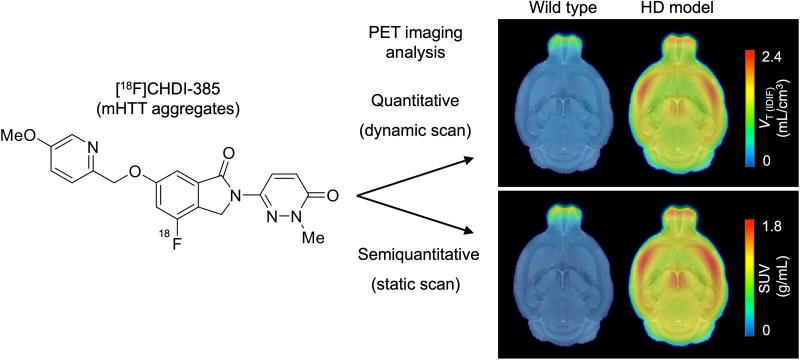

Huntington disease (HD) is a progressive genetic neurologic disorder caused by an expanded trinucleotide (cytosine–adenine–guanine) repeat in exon 1 of the huntingtin gene (*HTT*) that expresses a toxic mutant huntingtin protein (mHTT) (1,2). Accumulation of the full-length mHTT, alongside mHTT fragments, is associated with cellular dysfunction and the loss of neurons (3,4). This causative effect of mHTT and consequent pathophysiologic dysfunctions has led to therapeutic interventional strategies aimed at reducing mHTT levels, with some already in clinical trials (5).

Although mHTT quantification in peripheral compartments, such as cerebrospinal fluid or plasma, has been developed as a surrogate measure of mHTT levels for brain-targeting therapeutics (6–8), there is currently no noninvasive method to directly measure mHTT in the brain. PET imaging of mHTT aggregates offers a solution to evaluate the therapeutic potential of mHTT-lowering therapies on a regional level within the brain.

We previously reported on the evaluation of several radiotracers for mHTT imaging in preclinical imaging studies, namely [^11^C]CHDI-626 (9,10), [^11^C]CHDI-180R (11–14), [^18^F]CHDI-650 (15,16), and [^11^C]CHDI-009R (17,18). [^11^C]CHDI-626 and [^11^C]CHDI-180R progressed to first-in-human investigations (10,11,19), but clinical use of both has been discontinued. [^11^C]CHDI-626 evaluation was halted because of accumulation of a radiometabolite in the brain (20), and [^11^C]CHDI-180R assessment was paused because of high intersubject variability (21). Our second generation of mHTT aggregate radioligands included the first ^18^F-labeled radioligand, [^18^F]CHDI-650 (15), which displayed superior PET signal differentiation between heterozygous and wild-type zQ175DN mice (16) but limited quantitative reproducibility with poor test–retest reliability (16). In contrast, [^11^C]CHDI-009R was a highly promising radioligand (17), which led to the development of a structurally similar ligand [^18^F]CHDI-00905385 (abbreviated [^18^F]CHDI-385), that has a similar affinity and selectivity toward mHTT aggregates as [^11^C]CHDI-009R ([^18^F]CHDI-385 maximum binding capacity, 467.3; [^11^C]CHDI-009R maximum binding capacity, 448). The longer half-life of ^18^F (109.8 min) and the efficient labeling process of [^18^F]CHDI-385 suggest potential high practicability and clinical translatability (18).

We report here the in vivo evaluation of [^18^F]CHDI-385 in the zQ175DN knockin HD mouse model by analyzing in vivo plasma and brain stability and by characterizing the in vivo kinetic properties, including test–retest reliability. We evaluated both quantitative and semiquantitative methods to assess the feasibility of detecting phenotypic differences at 3 and 9 mo between heterozygous zQ175DN mice and their wild-type littermates.

## MATERIALS AND METHODS

### Animals

Forty-eight 3-mo-old and 48 9-mo-old male zQ175DN mice (C57BL/6J background) were obtained from Jackson Laboratories (22,23). Each group consisted of 24 heterozygous mice and 24 wild-type littermates. Before shipping, all animals were screened for sporadic congenital portosystemic shunt that occurs in C57BL/6J mice (24). Mice were shunt-free and given at least 1 wk to acclimate after their arrival before the start of any procedure. Mice were single-housed in ventilated cages under a 12-h light–dark cycle and kept in a temperature- and humidity-controlled environment with unrestricted access to food and water. Experiments followed the European Committee recommendations (decree 2010/63/CEE) and were approved by the Ethical Committee for Animal Testing (ECD 2019-39 and 2022-81) at the University of Antwerp. Mice characteristics are summarized in Supplemental Table 1 (test–retest analysis: 7 heterozygous, 9 wild-type), Supplemental Table 2 (9-mo cohort: 17 heterozygous, 19 wild-type), and Supplemental Table 3 (3-mo cohort: 23 heterozygous, 22 wild-type), available at http://jnm.snmjournals.org.

### Radioligand Synthesis

[^18^F]CHDI-385 was prepared using an automated radiosynthesis module (AllInOne; Trasis). The radiosynthesis protocol followed the steps for copper-mediated radiofluorination described by Lahdenpohja et al. (25). A detailed description of the procedure is provided in the supplemental materials.

Radiochemical purity was greater than 99%, and molar activity was 138.96 ± 27.14 GBq/µmol at the end of synthesis (*n* = 16). The average decay-corrected radiochemical yield was 17.50 ± 7.30% at the end of synthesis.

### Radiometabolite Analysis

Analyses of radiometabolite profiles of plasma and brain samples of mice given [^18^F]CHDI-385 via lateral tail vein injection were performed at 5, 15, 30, 60 and 90 min after injection (mean injected activity, wild-type: 4.12 ± 0.33 MBq in 200 μL; heterozygous: 4.11 ± 0.34 MBq in 200 μL; *n* = 3 per genotype and time point). The procedure was performed as previously reported (13,16) and is described in full in the supplemental materials. Deproteinized plasma or brain samples were counted in a γ-counter (extraction efficiencies of 91.4 ± 1.8% in plasma and 97.5 ± 0.8% in brain). The preconditioned reverse-phase high-performance liquid chromatography system (Kinetex EVO C18, 5 µm, 150 × 4.6 mm; Phenomenex) with a Phenomenex security guard precolumn used an elution phase consisting of 0.05 M NaOAc buffer (pH 5.5) and acetonitrile at a flow rate of 1 mL/min as follows: 67:33 (v/v) for 0–7 min, 33%–90% for 7–14 min, 10:90 (v/v) for 14–15 min, and 90%–33% for 15–19 min). Reverse-phase high-performance liquid chromatography fractions were collected at 0.5-min intervals for 12 min. The control procedure (spiking blood and brain with 185 kBq of radiotracer) was identical to that used for the other samples and confirmed that no degradation of the radioligand occurred during the work-up in plasma (94.3 ± 0.2% of intact tracer) and brain (93.9 ± 0.4% of intact tracer), as shown in Supplemental Figures 1A and 1B, Supplemental Table 4 at 0 min, and the radiochromatograms in Supplemental Figures 1C and 1D.

### PET Imaging Acquisition

Dynamic PET/CT images were acquired on Inveon microPET and CT scanners (Siemens Healthineers). Animal preparation was performed as previously described (26) using isoflurane anesthesia (5% induction; 1.5% maintenance). Animal and dosing information for both wild-type and heterozygous mice of all paradigms are provided in Supplemental Tables 1–3.

PET data were acquired in list mode. After the microPET scan, a 10-min CT scan (80 kV/500 μA) was performed for attenuation correction.

Acquired PET data were reconstructed into 45 frames of increasing duration (12 × 10 s, 3 × 20 s, 3 × 30 s, 3 × 60 s, 3 × 150 s, and 21 × 300 s), with a total scan length of 120 min. Images were reconstructed using a list-mode, iterative reconstruction in 8 iterations and 16 subsets of the 3-dimensional ordered-subset expectation maximization algorithm with proprietary spatially variant resolution modeling (27). Normalization, dead-time, and CT-based attenuation corrections were applied, and PET image frames were reconstructed on a 128 × 128 × 159 grid with 0.776 × 0.776 × 0.796 mm^3^ voxels. SUV and SUV images were derived from 100–120 min average interval of each individual scan (static scan).

### Image Processing and Analysis

Image processing and analysis were performed as previously described (10) with PMOD 3.6 software (PMOD Technologies); a detailed description is presented in the supplemental materials (10,13,16,28–30). For spatial normalization of the PET/CT images, brain normalization of the CT image to a CT template was performed as previously described (29) using volumes of interest adapted from the Waxholm atlas (13). Regional brain time–activity curves were extracted for the striatum, motor cortex, hippocampus, thalamus, and cerebellum. Cardiac extraction of the image-derived input function (IDIF) was performed as previously described (16). To observe the heart boundary with the liver, the dynamic PET image was averaged from 90 to 120 min, and the IDIF volume of interest was adjusted accordingly.

A consistent decrease in *V*_T(IDIF)_ values was observed with increasing injected mass of the isotopically stable compound (Supplemental Fig. 2); thus, the upper limit for mass injection was set at 1 µg/kg for practical considerations (molar activity) and to avoid the declining *V*_T(IDIF)_ values associated with increasing injected mass.

SUV and SUV images were based on the average of the 100–120 min range of each individual scan and scaled by the ratio of injected dose-to-body weight (30). Other time intervals were considered for semiquantitative analysis and demonstrated equally good agreement with Logan *V*_T(IDIF)_ values (Supplemental Fig. 3). However, the time interval of 100–120 min could best discriminate between genotypes (Supplemental Table 5) and was therefore chosen for further analysis.

### Statistical Analysis

The Shapiro–Wilk test revealed that in vivo data and in vitro data were normally distributed. Biexponential fit and linear fit were used to estimate intact radioligand and plasma-to-whole-blood ratio curves, respectively. Unpaired *t* tests were performed to investigate differences between genotypes in dosing parameters for scan acquisition. Paired *t* tests were performed to investigate differences between test and retest in dosing parameters for scan acquisition.

Pearson correlation with simple linear regression was applied to *V*_T(IDIF)_ values from 2-tissue compartmental model and Logan graphical analysis for model verification and to *V*_T(IDIF)_ values and SUV for analysis agreement. Spearman correlation with interpolation (sigmoidal, 4PL, X = log_[concentration]_) was applied to injected masses and *V*_T(IDIF)_ values from Logan graphical analysis to estimate the mass dose limit. Regular 2-way ANOVA with post hoc Bonferroni adjustment for multiple comparisons was applied to analyze *V*_T(IDIF)_ differences between genotypes in the investigated brain regions.

The individual relative test–retest variability (rTRV) was calculated as follows:

$$\frac{\text{test}-\text{retest}}{\text{average}\;(\text{test},\text{retest})}.$$ Eq. 1

The individual absolute test–retest variability (aTRV) was calculated using the following equation:

$$\left|\frac{\text{test}-\text{retest}}{\text{average}\;\left(\text{test},\text{retest}\right)}\right|.$$ Eq. 2

In addition, Bland–Altman analysis was performed to assess the agreement between measures on an individual level, with the given difference in percentage calculated with the same formula as rTRV. All statistical analyses were performed with Prism version 10.4.1 (GraphPad). Furthermore, the intraclass correlation coefficient (ICC) for the test–retest variability study was calculated with JMP Pro 17 software. Group × time point interaction was set as a fixed effect. Animal and time point, as well as the interaction of animal × group and animal × time point, were estimated as random effects. The animal × group and animal × time point interactions did not affect ICC calculation. The ICC was reported as the ratio of the animal variance component for the effect to the sum of positive variance components. One-tailed power analyses were performed in G*Power (v3.1.9.6) with significance (*α*) set at 0.05 and confidence (*β*) set at 80%. Data are represented as mean ± SD unless otherwise specified. All tests were 2-tailed unless stated otherwise. A *P* of less than 0.05 was considered statistically significant.

## RESULTS

The PET datasets generated or analyzed in this study are available in the OpenNeuro repository (https://doi.org/10.18112/openneuro.ds006691.v1.0.0).

### Stability of [^18^F]CHDI-385

**FIGURE 1.**
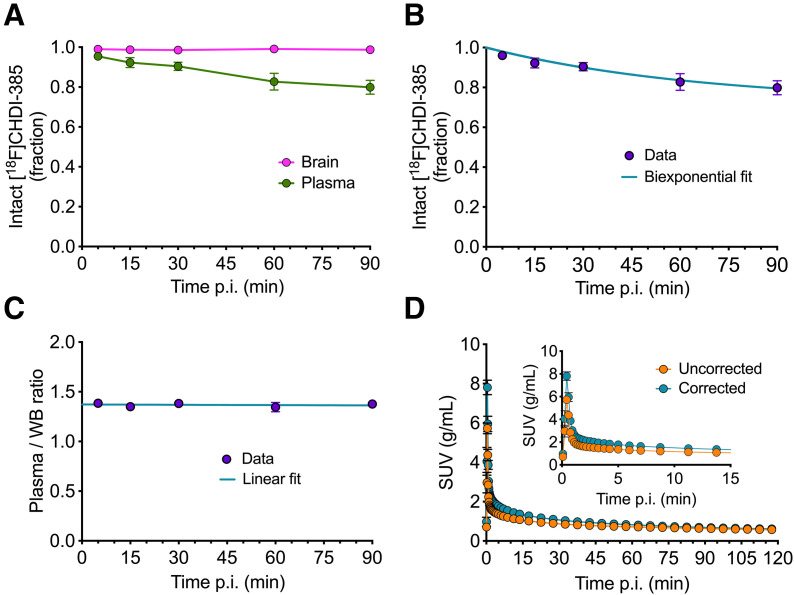
Radiometabolite analysis and IDIF correction. (A) Brain and plasma [^18^F]CHDI-385 intact radioligand for population-based data. (B) Fraction of intact [^18^F]CHDI-385 with biexponential fit applied for plasma data. (C) Plasma-to-whole-blood ratio with linear fit. (D) Averaged uncorrected and corrected IDIF SUV curves over 120-min scan time. p.i. = postinjection; WB = whole blood.

The radiometabolite analysis of [^18^F]CHDI-385 showed 79.9 ± 3.5% and 98.7 ± 0.7% intact parent radioligand remaining in plasma and brain samples, respectively, at 90 min after injection (Fig. 1A), with at least 2 polar radiometabolites identified in plasma (Supplemental Fig. 1). Because [^18^F]CHDI-385 displayed similar plasma radiometabolite profiles in wild-type and heterozygous zQ175DN mice (Supplemental Fig. 1; Supplemental Table 4), the combined radiometabolite data were used to generate metabolite-corrected IDIFs. The biexponential fit of the intact radioligand in plasma (Fig. 1B) and the linear fit of plasma-to-whole blood ratio (Fig. 1C) were used to determine the plasma metabolite-corrected input functions for kinetic modeling (Fig. 1D).

### Kinetic Modeling and Quantification of [^18^F]CHDI-385

**FIGURE 2.**
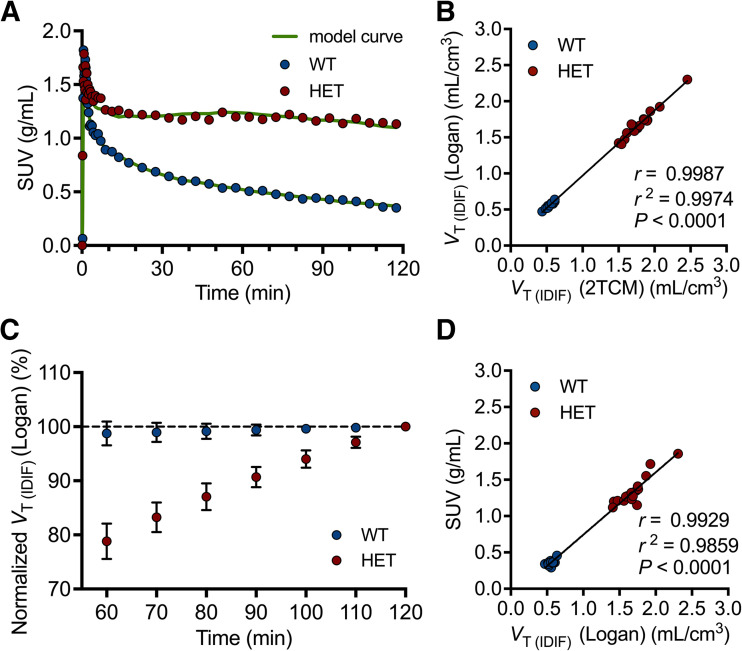
[^18^F]CHDI-385 kinetic modeling in wild-type (WT) and heterozygous (HET) mice. (A) Exemplary striatal SUV time–activity curves with 2-tissue compartmental model fit. (B) Pearson correlation of 2-tissue compartmental model and Logan graphical analysis. (C) *V*_T(IDIF)_ time stability based on *V*_T(IDIF)_ values averaged for striatum, motor cortex, hippocampus, thalamus, and cerebellum. (D) Pearson correlation of *V*_T(IDIF)_ values based on 120-min scan and SUVs derived from interval of 100–120 min.

Kinetic modeling based on striatal time–activity curves for wild-type and heterozygous mice revealed that a 2-tissue compartmental model was adequate (Fig. 2A), which agreed with Logan graphical analysis (*r^2^* = 0.997; *P* < 0.0001) (Fig. 2B). Hence, Logan graphical analysis was considered suitable to quantify [^18^F]CHDI-385 binding in mice.

Since 120 min is a demanding scan duration, a *V*_T(IDIF)_ time–stability analysis was performed to investigate whether shorter acquisition times may be feasible. Although no effect was observed in wild-type mice, the *V*_T(IDIF)_ in heterozygous mice continuously decreased with reduced scan acquisition time (78.8 ± 3.3% with 60-min acquisition), indicating that shorter scans are not recommended to avoid underestimating any phenotypic effect (Fig. 2C). Alternatively, we investigated the semiquantitative analysis using the time interval of 100–120 min after injection. As shown in Figure 2D, excellent agreement between *V*_T(IDIF)_ and SUV was observed (striatum, *r^2^* = 0.986; *P* < 0.0001), supporting the application of SUV for the measurement of [^18^F]CHDI-385 uptake in mice.

### Test–Retest Variability Analysis of [^18^F]CHDI-385

**FIGURE 3.**
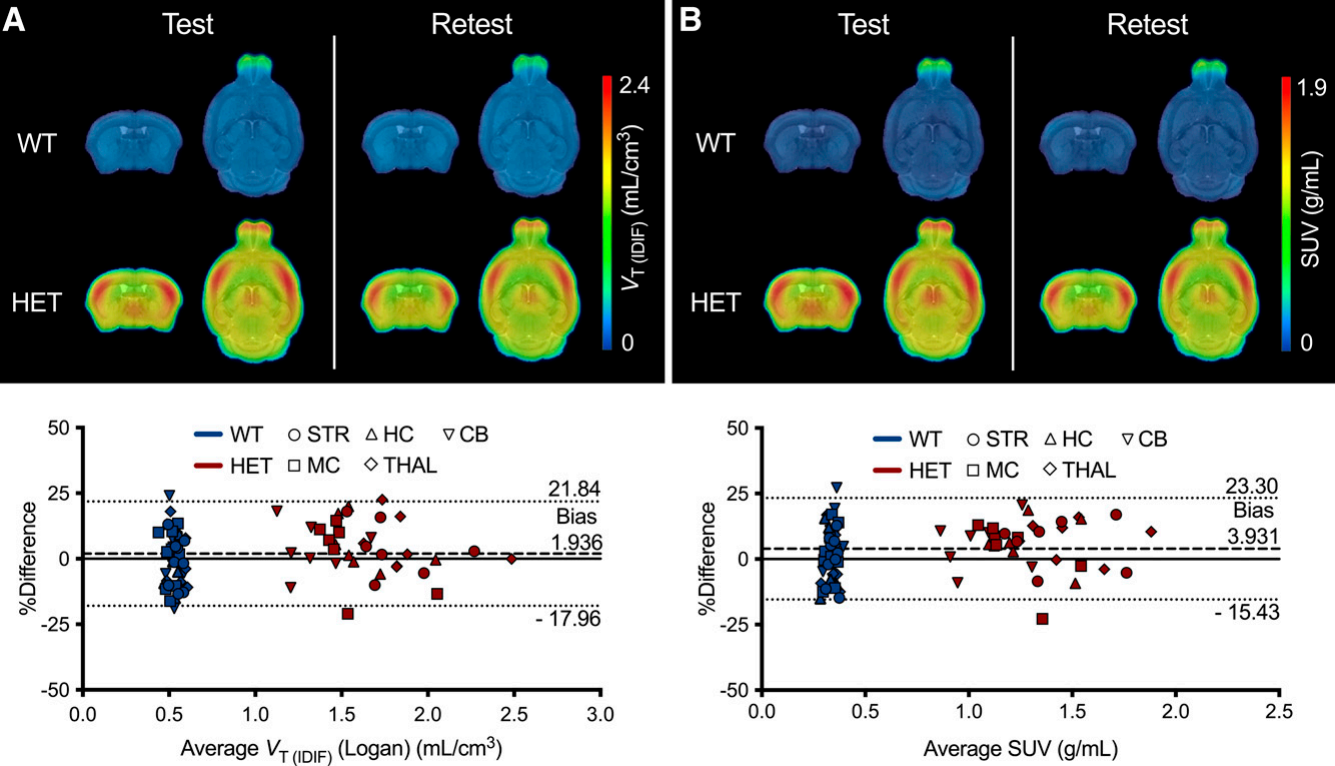
[^18^F]CHDI-385 test–retest variability analysis at 9 mo. Group-averaged *V*_T(IDIF)_ parametric maps (A) and group-averaged SUV (100–120 min) images (B) for wild-type (WT) and heterozygous (HET) zQ175DN mice at test and retest. Bland–Altman plots display level of agreement between test and retest using Logan graphical analysis (A) and semiquantitative analysis (B). CB = cerebellum; HC = hippocampus; MC = motor cortex; STR = striatum; THAL = thalamus.

The reliability of [^18^F]CHDI-385 was estimated in a test–retest paradigm. Neither the parametric *V*_T(IDIF)_ maps (Fig. 3A) nor the SUV images (Fig. 3B) revealed any obvious differences for either genotype. In Figure 3A, the Bland–Altman plot for all 5 brain regions based on *V*_T(IDIF)_ demonstrated close-range limits of agreement (−17.96% and 21.84%). The even distribution in *V*_T(IDIF)_ changes was also highlighted through the low rTRV values (ranging from −1.4 ± 9.5% and 5.8 ± 9.9%) and aTRV values (ranging from 7.4 ± 8.1% to 11.6 ± 5.6%) (Table 1). In addition, the ICC, used to classify the tracer variability, displayed a good to excellent test–retest agreement (0.62–0.78; Table 1). Similarly, SUV (semiquantitative analysis) demonstrated comparable high levels of agreement in the Bland–Altman plot (−15.43% and 23.30%; Fig. 3B), rTRV (range, 0.8 ± 9.1% to 7.7 ± 7.3%), and aTRV (range, 7.1 ± 5.2% and 10.6 ± 8.5%) (Table 1). The ICC demonstrated good to excellent test–retest agreement for the semiquantitative analysis (0.65–0.80; Table 1). In addition, *V*_T(IDIF)_ and SUV displayed low bias (1.936% and 3.931%, respectively).

**TABLE 1.** [^18^F]CHDI-385 Test–Retest Variability at 9 Months

	Quantitative*					Semiquantitative^†^				
	rTRV		aTRV			rTRV		aTRV		
Brain region	WT	HET	WT	HET	ICC	WT	HET	WT	HET	ICC
Striatum	−1.2 ± 9.3	3.9 ± 10.3	7.6 ± 4.9	8.4 ± 6.4	0.77	0.8 ± 9.1	6.3 ± 9.6	7.1 ± 5.2	10.2 ± 4.2	0.78
Motor cortex	0.5 ± 10.7	1.7 ± 13.5	8.9 ± 5.0	11.6 ± 5.6	0.62	2.0 ± 10.0	3.1 ± 12.5	7.6 ± 6.4	10.4 ± 6.5	0.65
Hippocampus	−1.4 ± 9.5	5.3 ± 9.8	7.7 ± 5.0	7.4 ± 8.1	0.73	0.8 ± 10.4	6.4 ± 9.0	8.4 ± 5.4	9.0 ± 5.9	0.73
Thalamus	0.7 ± 9.8	5.8 ± 9.9	8.0 ± 5.0	7.5 ± 8.5	0.78	1.3 ± 10.1	7.7 ± 7.3	8.6 ± 4.6	8.9 ± 5.5	0.80
Cerebellum	2.5 ± 11.9	4.0 ± 9.6	9.2 ± 7.3	7.6 ± 6.6	0.75	7.5 ± 11.7	5.6 ± 10.0	10.6 ± 8.5	9.0 ± 6.4	0.74

* Calculated using *V*_T(IDIF)_ with Logan graphical analysis.

† Calculated using SUV.

WT = wild-type; HET = heterozygous.

Data are represented as mean ± SD in percentage.

### [^18^F]CHDI-385 Quantification Confirmed Phenotypic Differences

**FIGURE 4.**
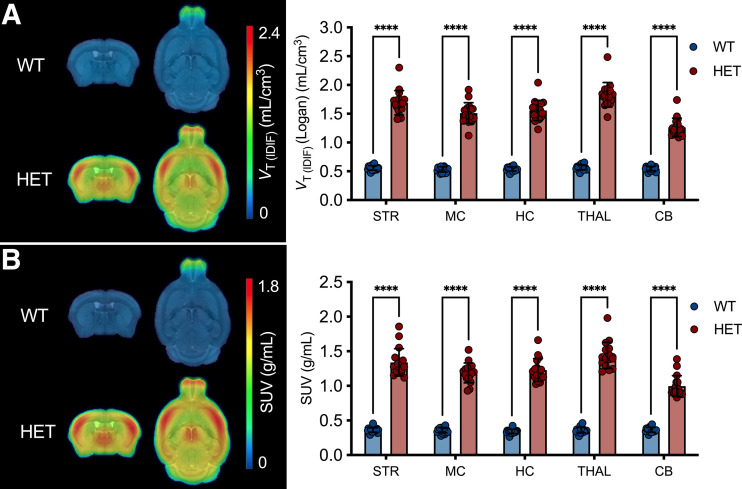
[^18^F]CHDI-385 mHTT aggregate signal at 9 mo. Group-averaged *V*_T(IDIF)_ parametric maps (A) and SUV images (B) for wild-type (WT) and heterozygous (HET) zQ175DN mice. Significant differences were found between genotypes in *V*_T(IDIF)_ estimations (A) and semiquantitative analysis (B). CB = cerebellum; HC = hippocampus; MC = motor cortex; STR = striatum; THAL = thalamus.

The phenotypic differences at 9 mo in [^18^F]CHDI-385 signal intensity and distribution are visualized in Figure 4. Two-way ANOVA revealed a significant genotype effect (*V*_T(IDIF)_: *F*_(1,170)_ = 2622, *P* < 0.0001; SUV: *F*_(1,170)_ = 2421, *P* < 0.0001). Bonferroni post hoc analysis demonstrated significantly increased values in heterozygous mice compared with their wild-type littermates in all tested brain regions, regardless of the metric used (*P* < 0.0001) (Fig. 4). *V*_T(IDIF)_ differences ranged from 130.7 ± 8.1% in the cerebellum to 221.3 ± 7.8% in the thalamus, and SUV differences ranged from 173.4 ± 11.0% in the cerebellum to 301.4 ± 11.2% in thalamus (Supplemental Table 6).

**FIGURE 5.**
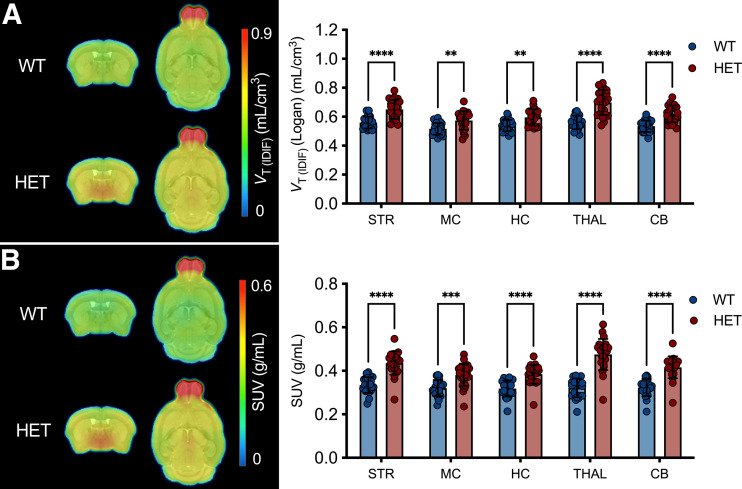
[^18^F]CHDI-385 mHTT aggregate signal at 3 mo. Group-averaged *V*_T(IDIF)_ parametric maps (A) and SUV images (B) for wild-type (WT) and heterozygous (HET) zQ175DN mice. Significant differences were found between genotypes in *V*_T(IDIF)_ estimations (A) and semiquantitative analysis (B). CB = cerebellum; HC = hippocampus; MC = motor cortex; STR = striatum; THAL = thalamus.

Additionally, [^18^F]CHDI-385 was used to visualize phenotypic differences at 3 mo (Fig. 5), with a high level of agreement between quantitative and semiquantitative analyses in combination with the 9-mo data (striatum, *r^2^* = 0.982; *P* < 0.0001; Supplemental Fig. 4). Two-way ANOVA revealed a significant genotype effect based on *V*_T(IDIF)_ (*F*_(1,215)_ = 131.0, *P* < 0.0001) and SUV (*F*_(1,215)_ = 219.6, *P* < 0.0001). Bonferroni post hoc analysis depicted statistically significant differences between genotypes (*P* < 0.0001) that ranged from 10.6 ± 3.1% to 24.6 ± 3.0% for *V*_T(IDIF)_ and 18.7 ± 4.5% to 47.4 ± 4.4% for SUV in all tested regions (Fig. 5; Supplemental Table 7).

### [^18^F]CHDI-385 Power Analysis

We used a power analysis (*α* = 0.05, *β* = 80%) to estimate the minimal sample size required per group in interventional studies. The effect size of [^18^F]CHDI-385 was reported as Cohen *d* for striatum at 9 mo (*V*_T(IDIF)_, 7.48; SUV, 6.85) and 3 mo (*V*_T(IDIF)_, 1.58; SUV, 2.43). Thus, [^18^F]CHDI-385 could detect a 20% difference between groups at 9 mo with small sample sizes for both metrics (*V*_T(IDIF)_, *n* = 12; SUV, *n* = 14). At 3 mo, assuming a 50% group difference, SUV would require a smaller sample size (*V*_T(IDIF)_, *n* = 31; SUV, *n* = 11; Supplemental Table 8).

## DISCUSSION

Our study characterized the in vivo performance of [^18^F]CHDI-385 in quantifying mHTT aggregates. [^18^F]CHDI-385 is of special interest because it shares a similar structure with [^11^C]CHDI-009R—which demonstrated high selectivity (18) and applicability in quantifying mHTT aggregates (17)—but has the advantage of an ^18^F radioisotope. In autoradiography studies of homozygous zQ175DN mice, both [^3^H]CHDI-009R and [^3^H]CHDI-385 exhibited increased specific binding compared with the previously validated [^3^H]CHDI-180 (18), a high-performing mHTT aggregate radioligand (13). Furthermore, [^18^F]CHDI-385 revealed similar in vivo properties, such as high stability in the brain and plasma, as well as a suitable brain retention time with reversible kinetics as observed with [^11^C]CHDI-009R (17). The quantitative analysis with *V*_T(IDIF)_ values suggested that [^18^F]CHDI-385 will discriminate genotypes at early and later ages, similar to previously described mHTT aggregate radioligands (13,16,17). [^18^F]CHDI-385, however, is the first ^18^F-labeled radioligand to demonstrate a low test–retest variability that differs from [^18^F]CHDI-650, which showed a relatively high test–retest variability (16). Noteworthy, [^18^F]CHDI-385 necessitates a long dynamic acquisition time of 120 min to estimate *V*_T(IDIF)_, which poses challenges for acquisition throughput and animal welfare. To address these challenges, we considered the use of semiquantitative SUV; however, the semiquantitative nature of the SUV makes it susceptible to potential biases. Not using an input function means that, among other parameters, alterations in brain perfusion or body weight could affect SUV (30–32). Nevertheless, with a priori validation using a full kinetic model (33), we found that semiquantitative analysis may be a valid alternative. In the case of [^18^F]CHDI-385, we observed good agreement between quantitative and semiquantitative analysis and excellent reliability with both metrics, indicating the suitability of semiquantitative analysis for [^18^F]CHDI-385 administration in mice. Ultimately, this validity may allow for optimal use of resources and a highly accurate applicability of this radiotracer in a preclinical setting. This could be beneficial for preclinical therapeutic evaluations and to facilitate clinical translation to the multiple interventional strategies currently under investigation, including reducing somatic instability (34,35), lowering mHTT (13,36), or targeting other processes involved in the formation of mHTT aggregates (5). The power analysis, based on the expected range of mHTT suppression in clinical studies (∼50%) (13), further demonstrated that [^18^F]CHDI-385 is suitable for preclinical therapeutic studies with both early- and late-treatment paradigms. This finding is consistent with that for our previous mHTT radiotracers, which were shown to be sensitive in detecting mHTT aggregate levels in young mice when the mHTT aggregate load is low (13,16,17). The higher phenotypic effect of [^18^F]CHDI-385 and the lower SD resulted in satisfactory Cohen *d* values. Taken together, the data suggest that [^18^F]CHDI-385 is an excellent candidate for preclinical evaluation of therapeutics aimed at decreasing the mHTT aggregate load and that it has adequate PET properties (18) to warrant clinical evaluation.

## CONCLUSION

[^18^F]CHDI-385 is a highly stable radiotracer with good to excellent reproducibility that can detect significant phenotypic differences at early ages in the zQ175DN HD mouse model. These features, in combination with the validated use of semiquantitative methods, present favorable properties for its application, especially in therapeutic studies. These findings suggest [^18^F]CHDI-385 is a suitable radioligand to image mHTT aggregates in mice and that clinical investigation is warranted.

## DISCLOSURE

This work was funded by CHDI Foundation, Inc.—a nonprofit biomedical research organization exclusively dedicated to collaboratively developing therapeutics that will substantially improve the lives of HD-affected individuals—under agreement number A-11627. Celia Dominguez, Robert Doot, Vinod Khetarpal, Jonathan Bard, and Longbin Liu are employed by CHDI Management, Inc., the company that manages the scientific activities of CHDI Foundation, Inc. No other potential conflict of interest relevant to this article was reported.

## Notes

KEY POINTS **QUESTION:** Can [^18^F]CHDI-385 PET imaging quantify mHTT aggregates in an in vivo mouse model of HD? **PERTINENT FINDINGS:** Preclinical assessment of [^18^F]CHDI-385 demonstrated desirable in vivo properties for both quantitative and semiquantitative measurement using the zQ175DN mouse model of HD. **IMPLICATIONS FOR PATIENT CARE:** The performance of [^18^F]CHDI-385 holds promise for accurate preclinical evaluation of therapeutic interventions, and clinical evaluation is warranted.
